# Immune checkpoint inhibitors increase T cell immunity during SARS-CoV-2 infection

**DOI:** 10.1126/sciadv.abg4081

**Published:** 2021-08-18

**Authors:** Nader Yatim, Jeremy Boussier, Pauline Tetu, Nikaïa Smith, Timothée Bruel, Bruno Charbit, Laura Barnabei, Aurélien Corneau, Laetitia Da Meda, Clara Allayous, Barouyr Baroudjian, Majdi Jebali, Florian Herms, Ludivine Grzelak, Isabelle Staropoli, Vincent Calmettes, Jerome Hadjadj, Olivier Peyrony, Charles Cassius, Jerome LeGoff, Nora Kramkimel, Selim Aractingi, Magnus Fontes, Catherine Blanc, Frederic Rieux-Laucat, Olivier Schwartz, Benjamin Terrier, Darragh Duffy, Celeste Lebbé

**Affiliations:** 1 Translational Immunology Laboratory, Department of Immunology, Institut Pasteur, F-75015 Paris, France.; 2 Université de Paris, APHP Hôpital Saint-Louis, Dermatology Department, DMU ICARE, INSERM U-976, Paris, France.; 3 Sorbonne Université, AP-HP Hôpital Saint-Antoine, F-75012 Paris, France.; 4 Virus and Immunity Unit, Department of Virology, Institut Pasteur, CNRS UMR 3569, Paris, France.; 5 Vaccine Research Institute, Creteil, France.; 6 Institut Pasteur, Cytometry and Biomarkers UTechS, CRT, F-75015 Paris, France.; 7 Université de Paris, Laboratory of Immunogenetics of Pediatric Autoimmune Diseases, Imagine Institute, INSERM UMR 1163, F-75015 Paris, France.; 8 Sorbonne Université, Faculté de Médecine, UMS037, PASS, Plateforme de Cytométrie de la Pitié-Salpêtrière CyPS, F-75013 Paris, France.; 9 Université de Paris, APHP Hopital Cochin, Department of Dermatology, Paris, France.; 10 Université de Paris, APHP Hopital Cochin, Department of Internal Medicine, National Referral Center for Rare Systemic Autoimmune Diseases, Assistance Publique Hôpitaux de Paris-Centre (APHP-CUP), F-75014 Paris, France.; 11 APHP Hôpital Saint-Louis, Emergency Department, Paris, France.; 12 Université de Paris, INSERM, Equipe INSIGHT, U976, Virology, AP-HP, Hôpital Saint Louis, F-75010 Paris, France.; 13 Institut Roche, Boulogne-Billancourt, France.

## Abstract

The COVID-19 pandemic has spread worldwide, yet the role of antiviral T cell immunity during infection and the contribution of immune checkpoints remain unclear. By prospectively following a cohort of 292 patients with melanoma, half of which treated with immune checkpoint inhibitors (ICIs), we identified 15 patients with acute or convalescent COVID-19 and investigated their transcriptomic, proteomic, and cellular profiles. We found that ICI treatment was not associated with severe COVID-19 and did not alter the induction of inflammatory and type I interferon responses. In-depth phenotyping demonstrated expansion of CD8 effector memory T cells, enhanced T cell activation, and impaired plasmablast induction in ICI-treated COVID-19 patients. The evaluation of specific adaptive immunity in convalescent patients showed higher spike (S), nucleoprotein (N), and membrane (M) antigen-specific T cell responses and similar induction of spike-specific antibody responses. Our findings provide evidence that ICI during COVID-19 enhanced T cell immunity without exacerbating inflammation.

## Teaser

Immune checkpoint inhibitors for melanoma improve adaptive T cell immunity during COVID-19 without exacerbating inflammation.

## INTRODUCTION

Severe acute respiratory syndrome coronavirus 2 (SARS-CoV-2) emerged in December 2019 and has spread worldwide, causing the coronavirus disease 2019 (COVID-19) pandemic with around 5% of infected cases suffering severe disease and 1% lethality (*1*, *2*). The impact of COVID-19 in patients suffering from cancer and the contribution of different classes of cancer treatment to COVID-19 severity are still under investigation. Early studies demonstrated a higher disease severity and fatality rate (reaching 28%), most probably due to immunosuppression and increased comorbidities (*3*, *4*); in contrast, later retrospective and prospective cohorts reported no increased death rate (*5*–*8*). These discrepant findings may result from cohort heterogeneity, especially in cancer types, stages, and treatments.

COVID-19 severity is driven by an excessive inflammatory response to infection, resulting in lung tissue damage, acute respiratory distress syndrome, and ultimately respiratory failure and death. Similarly to what has been described in septic shock (*9*), this hyperinflammation may lead to a cellular immunity exhaustion state marked by the up-regulation of immune checkpoints such as programmed cell death protein 1 (PD-1) and T cell immunoglobulin and mucin domain-containing protein 3 (TIM-3) on the surface of T cells and natural killer cells (*10*, *11*). Studies indicate that most patients with severe COVID-19 display lymphopenia caused by excessive T cell apoptosis and up-regulation of exhaustion markers (*12*). Moreover, longitudinal studies demonstrated the appearance of SARS-CoV-2–reactive CD38^+^ HLA-DR^+^ CD8^+^ and CD4^+^ T cells a few days before disease recovery, which remained detectable during convalescence; a significant proportion of these cells displayed up-regulated markers of exhaustion (*13*–*15*). This has led to the proposal of at least three ongoing clinical trials evaluating PD-1 inhibitors in the treatment of COVID-19 (NCT04356508, NCT04333914, and NCT04268537). However, recent analysis reported conflicting results concerning the risk of severe COVID-19 in anti-PD-1–exposed patients with lung cancer, which may be attributed to cofounding risk factors (*16*–*18*). Hence, it remains an open question whether immune checkpoint inhibitors (ICIs) play a beneficial role by stimulating a robust antiviral adaptive immune response, or whether they are detrimental, by leading to an excessive inflammatory response resulting in organ damage and failure (*19*).

In the following study, we prospectively characterized the clinical expression of COVID-19 and monitored the development of anti–SARS-CoV-2 antibodies in a cohort of stage III and IV melanoma patients. ICI-treated melanoma patients with active disease or after recovery (convalescent) were included for deep immune profiling that comprised transcriptional, mass cytometry, and T cell restimulation assays, providing a phenotypical and functional immune signature of the PD-1/PDL-1 axis in COVID-19 pathogenesis (ICI cohort). We found that ICI treatment was not associated with severe COVID-19 nor did it exacerbate inflammation and provide evidence that it increases specific anti–SARS-CoV-2 T cell immunity.

## RESULTS

### Epidemiological and clinical characterization of a melanoma cohort during COVID-19 epidemic

From 2 March to 30 June 2020, among 292 patients with stage III or IV melanoma, 15 SARS-CoV-2 infections were identified [by RT-PCR (reverse transcription polymerase chain reaction) and/or serology]. Age was similar between the whole patient cohort [median, 61 years; interquartile range (IQR), 50 to 72] and the COVID-19 subgroup (median, 58 years; IQR, 53 to 64). Males represented 56.9% of the whole cohort but 80% of infected patients. Prognostic factors reported in COVID-19—such as body mass index > 30 (20% versus 13.7%), diabetes (26.7% versus 10.6%), and hypertension (53.3% versus 29.1%)—were in higher proportions in patients with COVID-19, albeit with no statistically significant difference. Active smoking history was more frequent in the whole population (17.8% versus 6.7%), while congestive heart failure, chronic obstructive pulmonary disease, and chronic kidney disease were infrequent in both groups (Table 1). The most common presenting symptom that led to PCR testing was cough (15.1%) followed by shortness of breath (11.3%), myalgia (11.6%), diarrhea (11.0%), and fever (9.9%) (Table 1).

**Table 1:** Baseline and COVID-19–related characteristics of a stage III and IV melanoma patient cohort. *N*, number of patients; %, percentage of patients; ECOG, Eastern Cooperative Oncology Group; BMI, body mass index; COPD, chronic obstructive pulmonary disease; ICU, intensive care unit.

	**All patients (*n* = 292)**	**Patients with COVID-19 (*n* = 15)**
**Patient characteristics**		
Median age (range)	61 years (50–72)	58 years (53–64)
Male, *n* (%)	166 (56.9%)	12 (80%)
ECOG 0-1, *N*(%)	207 (70.9%)	13 (87%)
	Unknown 23 (7.9%)	
**Comorbidities *n*(%)**		
BMI > 30	40 (13.7%)	3 (20.0%)
Active smoker	52 (17.8%)	1 (6.7%)
COPD	1 (0.3%)	0 (0%)
Diabetes	31 (10.6%)	4 (26.7%)
Hypertension	85 (29.1%)	8 (53.3%)
Congestive heart failure	9 (3.1%)	0 (0%)
Corticosteroids	34 (11.6%)	3 (20.0%)
Immunosuppressive agents	5 (1.7%)	0 (0%)
Chronic kidney disease	8 (2.7%)	0 (0%)
**COVID-19–related symptoms *n*(%)**		
Asthenia	66 (22.6%)	4 (26.7%)
Chills	12 (4.1%)	1 (6.7%)
Fever	29 (9.9%)	5 (33.3%)
Conjunctival congestion	12 (4.1%)	0 (0%)
Nasal congestion	44 (15.1%)	3 (20.0%)
Headache	44 (15.1%)	2 (13.3%)
Anosmia	17 (5.8%)	4 (26.7%)
Cough	44 (15.1%)	5 (33.3%)
Sore throat	13 (4.5%)	0 (0%)
Sputum production	15 (5.1%)	1 (6.7%)
Shortness of breath	33 (11.3%)	5 (33.3%)
Nausea	19 (6.5%)	1 (6.7%)
Vomiting	7 (2.4%)	0 (0%)
Diarrhea	32 (11.0%)	2 (13.3%)
Myalgia	34 (11.6%)	5 (33.3%)
Any of the symptoms above	132 (45.2%)	9 (60.0%)
**COVID-19–related explorations *n*(%)**		
Lung CT scan performed	39 (13.3%)	4 (26.7%)
Lung CT scan suggestive of COVID-19	6 (2.0%)	3 (20.0%)
SARS-CoV-2 RT-PCR performed	93 (31.7%)	6 (40.0%)
SARS-CoV-2–positive RT-PCR	6 (2.0%)	6 (40.0%)
SARS-CoV-2 serology performed	151 (51%)	14 (93.3%)
SARS-CoV-2–positive serology	13 (4.4%)	13/14 (92.8%)
**COVID-19–related outcomes *n*(%)**		
Hospitalization	4 (1.4%)	4 (26.7%)
ICU	0 (0%)	0 (0%)
Death	1 (0.3%)	1 (6.7%)
**Melanoma characteristics *n*(%)**		
Stage III, adjuvant therapy	84 (28.7%)	1 (6.7%)
Advanced melanoma	206 (70.5%)	14 (93.3%)
Stage I–II melanoma	2 (0.07%)	0 (0%)
**Melanoma therapy *n*(%)**		
Targeted therapy (BRAF ± MEK inhibitors)	60 (20.5%)	2 (13.3%)
Immunotherapy	147 (50.3%)	6 (40%)
Anti–PD-1 only	113 (38.7%)	3 (20%)
Anti–PD-1 + anti-CTLA4	34 (11.6%)	3 (20%)
Other treatment*	24 (8.2%)	1 (6.7%)
No active treatment†	29 (9.9%)	4 (26.6%)
Local treatment or no systemic treatment	32 (11.0%)	2 (13.3%)

*Ongoing other treatments included chemotherapy (dacarbazine or temozolomide) and patients included in interventional clinical trials.

†Patients that have received a systemic treatment more than 6 months before study inclusion.

SARS-CoV-2 PCR was performed in 93 melanoma symptomatic patients (31.7%) and was positive in 6 patients (2%). Serological testing of SARS-CoV-2–specific antibodies upon each patient visit to our center and regardless of the presence of symptoms was performed in 151 patients (51%). It was positive in 13 patients, allowing the estimation of 8.6% COVID-19 seroprevalence in our cohort. Among the 13 patients with SARS-CoV-2–positive serology, 6 did not present COVID-19 symptoms. Four of 15 COVID-19 patients (26.7%) required standard hospitalization, and 1 patient with associated chronic lymphoid leukemia presented a severe form defined by oxygen requirement of >3 liters/min and died of respiratory distress syndrome related to the infection. Across the whole cohort, most (50.3%) patients had ongoing immunotherapy consisting of anti-PD-1 alone (38.7%) or the combination of anti-PD-1 and anti-CTLA4 (11.6%). A total of 20.5% of patients had ongoing targeted therapy, and only 9.9% had no active treatment for at least 6 months. We noted a trend for a lower frequency of SARS-CoV-2 infection among stage III patients undergoing adjuvant therapy (1.2%) in comparison to the frequency of SARS-CoV-2 infection in advanced melanoma (6.8%; *P* = 0.06). A total of 73% of stage III patients (adjuvant setting) were treated with ICI in contrast to 42% of patients with advanced melanoma.

### Clinical and biological characteristics of the study population

We next examined the impact of ICI on the immune response during SARS-CoV-2 infection in patients with melanoma. Five COVID-19 ICI-treated patients and 14 ICI-treated noninfected patients were analyzed. The demographic and clinical characteristics of the patients are shown in table S1. Three patients were sampled twice during the active stage and the convalescent stage, one patient was sampled once during the active infection stage, and another patient was sampled once during the convalescent disease stage. COVID-19 patients’ samples were thus divided into two groups, active infection (*n* = 4) and convalescent disease (*n* = 4).

Patients with active infection were analyzed after a median duration of 7 days (IQR, 3 to 17) after disease onset, and the median interval from the last dose of immunotherapy to COVID-19 diagnosis was 33 days (IQR, 11 to 39). All patients had at least one controlled coexisting illness, mainly hypertension and type 2 diabetes, and had mild-to-moderate disease, with oxygen requirement of <3 liters/min in two patients. No patient later required admission to an intensive care unit nor the use of mechanical ventilation.

Nonmelanoma healthy donors and patients with mild-to-moderate disease from our previous study (*13*) were also analyzed as a control cohort for ICI treatment. Their clinical and demographic characteristics are shown in table S1 (control cohort).

Laboratory findings included elevated C-reactive protein (CRP) (median, 12.8 mg/liter; IQR, 4.6 to 17.3), lactate dehydrogenase (LDH) (median, 507 IU/liter; IQR, 419 to 810), and marked lymphopenia (fig. S1), as previously described in other cohorts (*14*). In comparison to the control cohort, ICI-treated patients had impaired lymphocyte counts at baseline and upon infection (fig. S1), in contrast to monocytes that were higher at both baseline and upon infection (fig. S1). Proportions of monocyte subgroups (classical, transitional, and nonclassical) were similar (fig. S2). Neutrophil and platelet counts were not affected by infection or treatment status (fig. S1).

### Immunological signature in the blood of ICI-treated patients with COVID-19

**Fig. 1:**
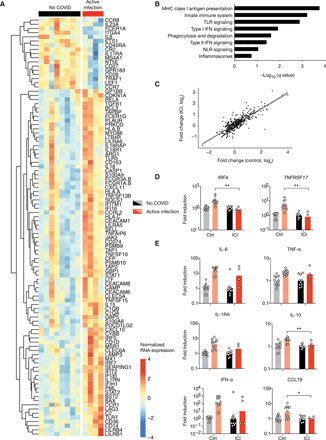
Patients treated with checkpoint inhibitors display typical transcriptomic and proteomic inflammatory response during active COVID-19. (**A** to **C**) Whole-blood transcriptomic profile of active COVID-19 patients and healthy controls from a cohort of nonmelanoma patients and a cohort of anti-PD-1–treated patients. (A) Heatmap representation of the 100 most differentially expressed genes between COVID-19–infected and uninfected patients of the anti-PD-1–treated cohort. (B) GSEA of pathways enriched during active COVID-19 in the PD-1–treated cohort. (C) RNA expression fold change between COVID-19 and uninfected patients in each cohort. Each point represents one gene. (**D**) RNA expression data from patients from both cohorts, normalized in each cohort to the mean of the uninfected group. (**E**) Proteomic data from patients from both cohorts, normalized as in (D). Each point corresponds to one patient, and bars represent mean value. Significance was determined using unpaired *t* tests. *P < 0.05 and **P < 0.01. MHC, major histocompatibility complex; TLR, Toll-like receptor; NLR, NOD-like receptor.

COVID-19 was reported to induce an excessive inflammatory response, with inflammation-related genes being increasingly expressed with disease severity. To assess the immunological transcriptional signature of active SARS-CoV-2 infection in ICI-treated patients, we analyzed the expression of 574 immune-related genes in whole blood from active COVID-19 patients compared to uninfected patients (Fig. 1A). Gene set enrichment analysis (GSEA; see Materials and Methods) revealed an up-regulation of genes belonging to innate immune and type I interferon (IFN) pathways (Fig. 1B) during active infection. ICI-treated patients displayed a similar signature in patients with mild and moderate COVID-19 compared to the control cohort [fig. S3 and (*20*)].

To further investigate potential differences in the COVID-19 gene signature between ICI-treated patients and the control cohort, we compared fold changes between infected and uninfected patients in each cohort and found a strong in-between-cohort correlation (Spearman’s *r* = 0.8, *P* < 0.0001; Fig. 1C). As an alternative strategy, we used the interaction term of a two-way analysis of variance (ANOVA) to unravel potential differences in COVID-19 gene signatures between control and ICI patients. We identified *IRF4* and *TNFRSF17* (Fig. 1D) as the sole two genes with a significant interaction after correction for multiple testing. There was no evidence for differential response in any other genes (all false discovery rates were >0.38). IRF4 is a key transcriptional factor essential for both the initial differentiation and the subsequent survival of plasmablasts (*21*), and TNFRSF17 [also named B cell maturation antigen (BCMA)] is a canonical plasmablast marker (*22*). Together, these results suggest that active SARS-CoV-2 infection in ICI-treated patients with melanoma triggers an inflammatory response that is similar to that of nonmelanoma patients.

We next measured protein plasma levels of key inflammatory cytokines. Interleukin-6 (IL-6) and tumor necrosis factor–α (TNF-α) were similarly induced during active infection, regardless of ICI treatment (Fig. 1E). Anti-inflammatory cytokines IL-1 receptor antagonist and IL-10 were both weakly induced in ICI-treated patients with COVID-19, in contrast to control COVID-19 (Fig. 1E). Comparing healthy donors to ICI-treated uninfected patients revealed higher baseline levels of IL-10 in ICI-treated patients with melanoma (fig. S4), suggesting a systemic anti-inflammatory state in ICI-treated patients, even in the absence of active infection.

Accumulating evidence points to a key role of type I IFN deficiency in the severity of COVID-19 (*20*, *23*, *24*). Plasma levels of IFN-α2 protein measured by Simoa digital enzyme-linked immunosorbent assay (ELISA) were higher in infected patients, regardless of ICI treatment (Fig. 1E). Consistent with protein levels, the IFN-stimulated gene (ISG) score, a validated score based on the mean expression of six ISGs defining a type I IFN signature (*20*), was also elevated in patients with COVID-19 (fig. S5). Nevertheless, we detected higher levels of basal ISG expression in ICI-treated patients (fig. S5), suggesting an active type I IFN signaling in these patients, in the absence of active infection. Overall, these data suggest that SARS-CoV-2 infection triggers a similar type I IFN response in ICI-treated and control patients.

COVID-19 has been associated with an influx of immune cells—including monocytes, neutrophils, and lymphocytes—to the infected lungs (*25*). We therefore quantified circulating chemokines involved in the trafficking of immune cells into inflammatory sites. We found no increase in CXCL2 and CCL2 (CC chemokine ligand 2) and CCL5 chemokines (fig. S6), in line with published reports at this disease stage (mild to moderate) (*14*, *20*) and supporting the results that ICI treatment did not exacerbate inflammatory responses. Unexpectedly, we found a significant increase in CCL19 plasma levels during infection that was absent in ICI-treated patients (Fig. 1E).

Together, these data indicate that SARS-CoV-2 infection induces typical transcriptional and proteomic signatures in ICI-treated patients, similar to what has been previously described in COVID-19 patients with mild-to-moderate disease. Differences have nevertheless been noted, such as increased basal IFN and a dampened induction of anti-inflammatory cytokines upon infection, which will require additional investigation.

### Immunophenotyping of COVID-19 ICI-treated patients with melanoma

**Fig. 2:**
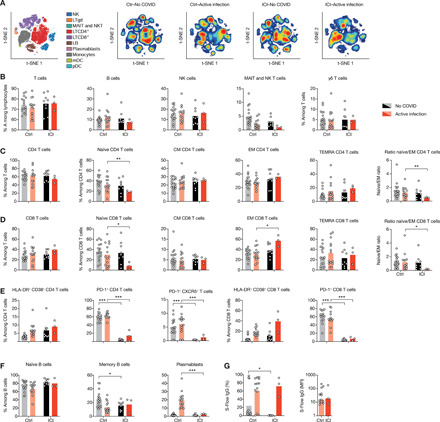
ICI-treated patients display an increased T cell activation profile. (**A**) viSNE map of blood leukocytes after exclusion of granulocytes, stained with 30 markers and measured with mass cytometry. Cells are automatically separated into spatially distinct subsets according to the combination of markers that they express (left). LTγδ, γδ T lymphocytes; MAIT, mucosal-associated invariant T cells; LB, B lymphocytes; NK, natural killer cells. viSNE map was then colored according to cell density in infected and uninfected patients in both cohorts. Red indicates the highest density of cells, and blue indicates the lowest. (**B**) Proportion of CD3^+^ T cells, CD19^+^ B cells, CD3^−^CD56^+^ NK cells, and MAIT cells among lymphocytes in peripheral blood. (**C** and **D**) Proportions of T cell subsets. (**E**) Analysis of the functional status of specific T cell subsets based on the expression of activation (CD38 and HLA-DR), exhaustion (PD-1), and Tfh markers (CXCR5^+^PD-1^+^). (**F**) Proportions of B cell subsets. (**G**) Quantification of SARS-CoV-2 spike (S)–specific IgG antibodies using the S-Flow assay. Left: Percentage of cells expressing S protein. Right: Normalized mean fluorescence intensity (MFI). Each point corresponds to one patient, and bars represent mean value. Significance was determined using unpaired *t* tests followed by Holm-Sidak correction for multiple testing. *P < 0.05, **P < 0.01, and ***P < 0.001.

To further characterize lymphocytopenia in ICI-treated patients with melanoma, blood was simultaneously collected for immune profiling. We used mass cytometry and performed visualization of *t*-distributed stochastic neighbor embedding (viSNE) to compare cell population densities according to melanoma and treatment (Fig. 2A and table S2). viSNE representation (Fig. 2A) and differentiated cell counts (fig. S7) showed a decrease in CD19^+^ B cells and CD3^+^ T cells, including all T cell subsets, which was more pronounced in ICI-treated patients, and with no major imbalance in the CD4^+^ and CD8^+^ T cell ratio (Fig. 2, B to D).

In comparison to control COVID-19 patients, we observed a significant decrease in the proportion of naïve CD8^+^ T cells in patients with COVID-19 that were treated with ICI, associated with a specific increase in the proportion of effector memory (EM) CD8^+^ T cells and resulting in a pronounced inversion of the naïve-to-EM cell ratio (Fig. 2D and fig. S7C). This expansion of the EM subset was less pronounced in CD4^+^ T cells (Fig. 2C and fig. S7B). Moreover, proportions of different T helper subsets (T_H_1, T_H_2, T_H_17, and regulatory T cells) remained unchanged (fig. S8).

We next assessed the functional status of T cells using markers of activation (CD38 and HLA-DR) and exhaustion (PD-1) (Fig. 2E). Both the CD4^+^ and CD8^+^ T cell populations were characterized by an increase in CD38^+^ HLA-DR^+^ activated T cells in all infected patients, with a more important increase in CD8^+^ T cells from patients treated with ICI (Fig. 2E). Notably, PD-1 detection was markedly diminished by ICI treatment, indicating lasting active checkpoint inhibition.

We next assessed the dynamics of different B cell subsets. Proportions of naïve and memory B cells were decreased in control infected patients, counterbalanced by an increase in plasmablasts (Fig. 2F). Unexpectedly, plasmablasts only modestly increased in the blood of ICI-treated patients (Fig. 2F), and this had no impact on anti–spike protein immunoglobulin G (IgG) antibody amounts (Fig. 2G). These results are consistent with the finding that ICI-treated patients had decreased expression of *IRF4* and *TNFRSF17* genes (Fig. 1D), both implicated in plasmablast differentiation and survival. Overall, these data suggested that ICI-treated patients exhibited features of increased T cell activation during SARS-CoV-2 infection.

### T cell–specific responses in ICI-treated patients with melanoma

Antigen-specific T cell responses and the production of neutralizing antibodies determine disease trajectory outcome during late-stage SARS-CoV-2 infection (*13*, *25*, *26*). We evaluated specific adaptive immune responses in ICI-treated convalescent patients in comparison to nonmelanoma convalescent patients (table S1). Patients were analyzed after a median duration of 42 days (IQR, 32 to 52) after disease onset, and median interval from the last dose of immunotherapy to COVID-19 diagnosis was 58 days (IQR, 28 to 73). All had displayed mild-to-moderate COVID-19 disease.

**Fig. 3:**
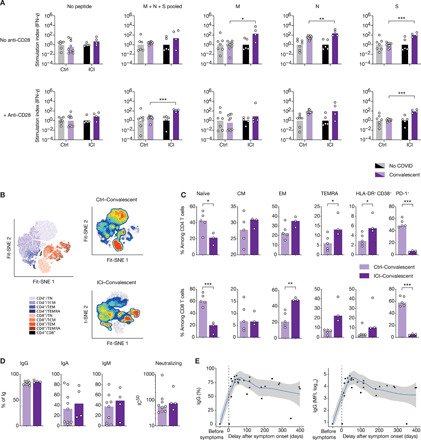
ICI treatment has a lasting impact on SARS-CoV-2 antiviral cellular immunity. (**A**) Whole blood was collected from COVID-19 convalescent patients and was stimulated with spike protein (S), nucleoprotein (NP), or membrane protein (M) peptide pools for 48 hours, and then supernatants were collected for IFN-γ measurement by digital ELISA. Stimulation index consists of IFN-γ concentrations normalized by the uninfected group of each cohort. (**B**) Fit-SNE map of lymphocytes from convalescent patients, measured with mass cytometry, map colored according to cell density. Red indicates the highest density of cells, and blue indicates the lowest. (**C**) Proportions of T cell subsets in convalescent patients of both cohorts. (**D**) Frequency of SARS-CoV-2 S–specific IgG^+^, IgA^+^, or IgM^+^ cells for each Ig subtype. (**E**) Longevity of SARS-CoV-2 S–specific IgG^+^ in ICI-treated patients, shown as percentage or MFI. Each point corresponds to one sample (*n* = 33), and bars represent mean value. Nonparametric regression curve was obtained using the LOESS (locally estimated scatterplot smoothing) method. Significance was determined using unpaired *t* tests. *P < 0.05, **P < 0.01, and ***P < 0.001. IC_50_, median inhibitory concentration.

Whole blood was collected and stimulated with spike protein (S), nucleoprotein (NP), or membrane protein (M) peptide pools for 48 hours, and supernatants were collected for IFN-γ measurement by digital ELISA. In line with our finding that ICI-treated patients had increased T cell activation during infection, ICI-treated convalescent patients demonstrated increased antigen-specific T response in comparison to nonmelanoma convalescent patients (Fig. 3A and fig. S9); this response was mainly driven by S and NP peptides. T cell phenotyping using mass cytometry and Fit-SNE analysis showed an altered density profile in both CD4^+^ and CD8^+^ T cell populations in ICI-treated convalescent patient in comparison to control convalescent patients (Fig. 3B and fig. S10). ICI-treated convalescent patients were characterized by a persistent decrease in the proportion of naïve CD4^+^ and CD8^+^ T cells (Fig. 3C), associated with an increase of both EM and TEMRA T cell subsets, whereas the central memory (CM) T cell subset remained stable (in comparison to the active infection phase). In contrast, proportions of CD38^+^ HLA-DR^+^ T cells returned to baseline levels (Fig. 3C). Analysis of two COVID-19 melanoma patients treated with targeted therapy (BRAF and MEK inhibitors) in convalescent stage demonstrated a phenotypic profile that was similar to nonmelanoma COVID-19 patients (fig. S11). In addition, ICI did not alter antibody levels nor their neutralizing activity (Fig. 3D and fig. S12A), and the longevity of anti-spike IgG and IgA antibodies was comparable in ICI-treated patients and nonmelanoma patients (Fig. 3E and fig. S12B) (*27*–*29*). Transcriptional analysis on whole blood collected from convalescent patients confirmed a persistent activated immunological state in comparison to control convalescent patients (fig. S13, A and B). Together, these data provide evidence that ICIs had a profound and prolonged impact on SARS-CoV-2 antiviral cellular immunity.

## DISCUSSION

The COVID-19 pandemic raised difficult challenges regarding the management of patients with cancer, the latter being considered as a high-risk population. However, it is not clear how treatment with ICI modulated SARS-CoV-2 infection susceptibility and disease outcome. By prospectively following a monocentric cohort of patients with melanoma, we identified 15 patients with COVID-19 that determined a 5% SARS-CoV-2 infection rate, with a quarter of the patients requiring hospitalization. These findings suggested that melanoma and ICI-treated patients were not a particularly vulnerable population—apart from coexisting susceptibilities such as age and other comorbidities. These preliminary results are in line with others (*17*, *30*) and support the safety of continued use of PD-1–blocking agents during the COVID-19 pandemic.

The impact of ICI on viral infections has been widely investigated in the context of chronic infections (*31*–*34*), yet how anti-PD-1 blockade affects cellular immunity during acute viral infection has been only sparsely studied (*35*). We found no major difference in cytokine and chemokine expression induced by ICI treatment, particularly in TNF-α and IL-6, which were previously shown to drive critical disease. We also found similar induction of IFN-α in both cohorts, a key cytokine that determines disease outcomes (*20*). Furthermore, we describe enhanced antigen-specific T cell responses in ICI-treated patients and increased expansion of EM CD8^+^ T cells during acute and convalescent phases.

Emerging evidence is challenging the concept of a hyperinflammatory state as being a driver of critical COVID-19, with recent reports demonstrating that hypercytokinemia is less marked in COVID-19 than in other critical conditions (*36*–*38*). These findings support alternative mechanisms of COVID-19–mediated critical disease, including the possibility that profound immunosuppression, through sustained quantitative and qualitative loss of CD4^+^ and CD8^+^ T cells, could be a key contributor of disease. It was shown that acute SARS-CoV-2 infection leads to rapid deficiency in T cell functionality and that specific CD8^+^ T cells are essential for viral control and better outcomes in mild infections (*26*, *39*). Here, we found that during active SARS-CoV-2 infection, patients undergoing treatment with ICI induced higher proportions of EM CD8^+^ T cells, a difference that persisted in convalescent patients with the additional expansion of TEMRA CD8^+^ T cells. While we were not able to evaluate the specificity of these T cell populations, we found that ICI-treated patients had an enhanced response to SARS-CoV-2 peptides, supporting the hypothesis that checkpoint inhibition enhanced CD8^+^ T cell immunity to SARS-CoV-2.

Intriguingly, recent findings reported that, although detectable and sustained, memory CD8^+^ T cells specific to SARS-CoV-2 were found at frequencies that were 10-fold lower than upon influenza A virus or Epstein-Barr virus infection (*40*). The authors suggested that SARS-CoV-2 infection limits both clonal expansion and differentiation of EM and CM subsets, with implications on clinical severity and vulnerability for future reinfection. Our data provide experimental evidence that treatment with anti-PD-1 agents may restore this defect and suggest that SARS-CoV-2 infection limits T cell expansion and differentiation in part via the up-regulation of exhaustion markers.

In contrast to the induction of effector T cells during infection, we found that ICI-treated patients did not increase the proportion of plasmablasts, the B cell “effectors.” Plasmablasts are rapidly induced upon viral infection, including SARS-CoV-2 infection (*25*), and are responsible for the production of the first antibody wave during acute infection. The mechanism by which PD-1 blocking agents inhibit the expansion of plasmablasts is unknown; it is possible that PD-1 blockade elicits the differentiation of plasma cells. PD-1 is highly expressed on T follicular helper (Tfh) cells, and the PDL1-PD-1 interaction is known to block the migration of Tfh into lymphoid follicles (*41*). Alleviating this blockade gives germinal center B cells a competitive advantage (*41*). Intriguingly, recent evidence supports a crucial role of Tfh during SARS-CoV-2 infection and suggests a pathogenic role for extrafollicular B cell responses (*42*). Despite this defect in plasmablasts, we found similar induction of SARS-CoV-2–specific antibodies during the active phase, which persisted in the convalescent phase. A longitudinal follow-up of antibody response for up to 1 year confirmed similar antibody longevity (Fig. 3E and fig. S12B). This observation warrants further evaluation and may provide important insights into the mechanisms underlying induction of antibodies during infection and their regulation by the PD-1 axis.

Our study has limitations. First, it is a monocentric, cross-sectional study built from a prospectively followed melanoma cohort, with a limited number of infected patients, particularly in the ICI-treated cohort. Conclusions regarding some of the immunological and the clinical impact of anti-PD-1 treatment on COVID-19 are to be taken with caution and require further validation with larger cohorts. Second, our control cohort (nonmelanoma non-ICI treatment) was extracted from an independent study performed simultaneously and using the same technological pipeline in another center. A cohort of untreated stage III or IV melanoma patients would have been an ideal, but challenging to form, control group. Third, the study was not designed as a longitudinal study, so few sequential measurements were available.

In summary, our data, in line with others (*17*, *30*), support the claim that treatment with anti–PD-1 agents does not pose a supplementary risk for patients with melanoma cancer during the COVID-19 pandemic. Careful evaluation of the benefits and risks balance, including individual risk factors, should be performed before each visit, and subsequent treatment choice should be performed. Moreover, immunological analysis suggests that ICIs do not exacerbate inflammation and may be beneficial in accelerating and amplifying antiviral T cell immunity in the context of acute infection, as well as establishing long-term immunity. This hypothesis warrants further evaluation and, if validated, may have important implication in strategies aiming to treat viral infection or to improve vaccination efficacy (*43*, *44*).

## MATERIALS AND METHODS

### Study design and patient population

Patients included in this study were identified via MelBase, a French clinical database with a biobank dedicated to the prospective follow-up of adult patients with melanoma. MelBase protocol was approved by the French ethics committee (CPP Ile-de-france XI, n°12027, 2012) and registered in the National Institutes of Health clinical trials database (NCT02828202). Written informed consent was obtained from all patients.

We prospectively collected clinical data based on a standardized clinical questionnaire designed to screen for possible symptoms suggestive of COVID-19, done routinely in all patients with melanoma (*n* = 292) treated by ICI or targeted therapy, in the adjuvant or metastatic settings, during the first French lockdown (17 March 2020), in Saint-Louis Hospital, onco-dermatology unit. All patients were contacted before their theoretical venue or during a teleconsultation with a standardized questionnaire screening for possible symptoms suggestive of COVID-19.

Patients presenting symptoms suggestive of SARS-CoV-2 infection identified via the survey were clinically evaluated for severity criteria, and nasal swabs for PCR testing were performed. Thoracic computerized tomography (CT) scan was performed at the physician’s discretion. Serum for measurement of SARS-CoV-2–specific antibody response was also collected from all patients during the epidemic period until 30 June 2020 and systematically upon each patient visit to our center, regardless of the presence of symptoms.

To understand the contribution of ICIs to the immune response to SARS-CoV-2 infection, blood from confirmed cases was collected for routine blood analysis and extensive immune profiling. Inclusion criteria for this subgroup of COVID-19–infected patients were as follows: age between 18 and 80 years old, infusion of PD-1 blockade antibody within the last 6 weeks before inclusion, diagnosis of COVID-19 according to the World Health Organization (WHO) interim guidance, and positive SARS-CoV-2 RT-PCR testing on nasopharyngeal swab. Patients with an additional malignant disease (including hematological and solid cancer) or with bacterial coinfection were excluded. Patients with a documented SARS-CoV-2 infection at more than 21 days from symptoms onset and with a negative SARS-CoV-2 RT-PCR test result were included as convalescent patients. Uninfected controls matched for age, sex, and treatment were also recruited among the melanoma cohort. Groups were therefore defined as melanoma controls (ICI–No COVID: uninfected ICI-treated patients with melanoma), melanoma with active COVID-19 (ICI–Active infection: ICI-treated melanoma patients with active disease), and melanoma convalescent patients (ICI–Conv: ICI-treated patients with melanoma during the convalescent phase after a proven mild-to-moderate disease). In addition, nonmelanoma patients were included as control samples and defined as controls (Ctrl–No COVID: uninfected healthy participants), active COVID-19 (Ctrl–Active infection: nonmelanoma patients with active disease), and convalescent patient (Ctrl–Conv: nonmelanoma patients during the convalescent phase after a proven mild disease). Data for nonmelanoma patients (uninfected and active infection) were collected from our previous study performed at Cochin Hospital (Paris, France).

Epidemiological, demographic, clinical, laboratory, treatment, and outcome data were extracted from electronic medical records using a standardized data collection form. Routine blood examinations were complete blood count, plasmatic biochemical tests (including renal and liver function, LDH, and electrolytes), CRP, and ferritin. Chest radiographs or CT scans were also performed at the physician’s discretion.

Laboratory confirmation of SARS-CoV-2 was performed at Saint-Louis Hospital, Virology Department, Paris, France. RT-PCR assays were performed in accordance with the protocol established by the WHO, Coronavirus disease (COVID-19) technical guidance: laboratory testing for 2019 nCoV in humans. The severity of COVID-19 was classified on the basis of the adaptation of the Sixth Revised Trial Version of the Novel Coronavirus Pneumonia Diagnosis and Treatment Guidance.

### Multiparameter phenotyping of peripheral blood leukocytes using mass cytometry

The Maxpar Direct Immune Profiling System (Fluidigm Inc., Canada) was used for high-dimensional immune profiling of whole blood, using a 30-marker antibody panel with the addition of two markers: anti-PD-1 conjugated to 175Lu (1 μg/μl concentration) and anti-Tim3 conjugated to 165Ho (1 μg/μl concentration). The list of antibodies and definition of cellular subsets used and staining protocols are summarized in (*20*) and table S2.

Cell events were acquired on the Helios mass cytometer and CyTOF software version 6.7.1014 (Fluidigm Inc., Canada) at the “Plateforme de Cytométrie de la Pitié-Salpetriere (CyPS).” An average of 400,000 events were acquired per sample. Mass cytometry standard files produced by the HELIOS were normalized using the CyTOF software v.6.7.1014. This method normalizes the data to a global standard determined for each log of EQ beads.

FCS3.0 files generated by the Helios were analyzed using GemStone software (Verity Software House, Topsham, ME), an automated analysis system. This system is integrated with dimensionality reduction mapping known as Cauchy enhanced nearest-neighbor stochastic embedding (Cen-se), which generates a visual display of high-dimensional data labeled with the major cell populations.

The multiparametric analysis of activation and immune checkpoint markers was performed on FlowJo, and the data generated were then analyzed using Tableau Desktop. For whole blood cell analysis, a viSNE analysis was performed (Cytobank Inc., Mountain View, CA, USA), mapping and integrating a total of 50,000 peripheral blood mononuclear cell samples analyzed for each group FCS file. Parameter viSNE maps were created with all markers. The settings used for the viSNE run were as follows: iterations (3000), perplexity (70), and theta (0.5). viSNE maps are presented as means of all samples in each category. For T cell analysis, a Fit-SNE analysis was performed (OMIQ, Santa Cruz, CA, USA), mapping and integrating a total of 60,000 T cell samples analyzed for each group FCS file. Parameter Fit-SNE maps were created with CD4, CD8, CCR7, CD45RA, CD45RO, CD28, CD38, HLA-DR, and CD27. The settings used for the Fit-SNE run were as follows: iterations (2000), perplexity (50), and theta (0.2). Fit-SNE maps are presented as means of all samples in each category.

### Gene expression analysis

As previously described (*20*, *45*), total mRNA was diluted with ribonuclease-free water at 20 ng/μl in 12 strips. We analyzed 100 ng (5 μl) of total RNA from each sample using the NanoString human immunology kit v2 according to the manufacturer’s instructions. Each sample was analyzed in a separate multiplexed reaction including in each eight negative probes and six serial concentrations of positive control probes. Data were imported into nSolver analysis software (version 4.0, NanoString) for quality checking and then exported as a table, and all subsequent analyses were performed using R (version 3.4.3 CRAN), using ggplot2 for plots. Background level was computed as mean + 2 SD of the negative control probes, for all samples. The housekeeping genes were selected from the 15 candidate control genes provided by NanoString, following the geNorm method (*46*). Briefly, after selection of genes with all values above the background level, for each two genes *j* ≠ *k*, pairwise variation coefficient *V_jk_* is defined as

$$V_{\mathrm{jk}}=sd_{i}({\text{log}}_{2}(\frac{a_{\mathrm{ij}}}{a_{\mathrm{ik}}}))$$

where *a_ij_* is the number of counts for the gene *j* in the sample *i*. The gene stability measure *M_j_* for control gene *j* is the arithmetic mean of all pairwise variations *V_jk_* for *k* ≠ *j*. *M_j_* evaluates the degree of correlation of gene *j* to other control genes (the smaller *M_j_* is, the more correlated gene *j* is to other control genes). Genes were ranked by increasing *M*, and to determine a threshold, the normalization factors NF*_n_* were computed for all *n* (defined as the geometric mean of the housekeeping gene counts) of each sample when considering the *n* genes with the lowest *M* as a housekeeping gene set. Correlations between consecutive normalization factors increased then decreased when adding the sixth gene with lowest *M*. This threshold was confirmed by studying the pairwise variation between consecutive NF*_n_*s. The final housekeeping gene set consisted of the following six genes: *TBP*, *TUBB*, *GUSB*, *POLR1B*, *SDHA*, and *ABCF1*. Normalization was performed as follows: The scaling factor for a sample was defined as the ratio of the average across all geometric means and the geometric mean of the sample. For each sample, all gene counts were multiplied by the corresponding scaling factor. After normalization, background level was recomputed as 54 normalized counts. Genes with no values above background levels as well as positive and negative control and housekeeping genes were removed from subsequent analyses. Normalized counts were log_10_-transformed for all subsequent analyses. Data from both cohorts were normalized using these housekeeping genes. As samples from the two cohorts were processed in separate batches, data from each cohort were normalized by the mean of the non–COVID-19 patients for cohort comparisons.

### Group comparisons, heatmaps, and GSEA

For each group comparison, genes with at least one value above background levels in one of the groups were tested. *t* tests, which are shown to be robust against non-normality, were performed to compare groups for each gene. GSEA plots were obtained by feeding the list of genes ordered by their *t* statistics to the GSEA algorithm (version 4.0.3, Broad Institute), along with a pathway dataset built from the NanoString Immunology panel version 2 annotation file. Parameters were set as follows: method, preranked gene list; number of permutations, 10,000; enrichment statistic, classic; minimum set size, 5; maximum set size, 500; and all other parameters as default. Heatmaps were generated with the 100 genes with the smallest *P* values, using pheatmap (package pheatmap), with data centered to 0 and scaled to unit variance for each gene.

To assess differential gene signatures upon SARS-CoV-2 infection in control versus ICI-treated patients and because RNA data did not clearly violate the homoscedasticity assumption, we performed, for each gene, a two-way ANOVA and reported the *P* value for the interaction term, followed by false discovery rate computation.

### Cytokine assays

Before protein analysis, plasma samples were treated in a P3 laboratory for viral decontamination using a protocol previously described for SARS-CoV, which we validated for SARS-CoV-2. Briefly, samples were treated with Triton X-100 (TX100) 1% (v/v) for 2 hours at room temperature (RT). IFN-α2, IFN-β, and IL-17A protein plasma concentrations were quantified by a Simoa triplex assay developed with Quanterix Homebrew kits as previously described (*13*). IL-6, TNF-α, and IL-10 were measured with a commercial triplex assay (Quanterix). The limits of detection of these assays were 2 fg/ml for IFN-α and 7 fg/ml for IFN-β. Additional plasma cytokines and chemokines were measured with a commercial Luminex multianalyte assay (Biotechne, R&D Systems).

### SARS-CoV-2–specific T cell restimulation assay

One hundred microliters of whole blood was incubated with 200 μl of TruCulture media (Myriad RBM) with overlapping peptides from SARS-CoV-2 proteins M, N, and S either individually or pooled (M + N + S) (Miltenyi Biotec) (2 μg/ml), with or without anti-CD28 (2 μg/ml) costimulation, for 48 hours at 37°C. After incubation, supernatants were collected and frozen at −80°C before IFN-γ quantification with Simoa ELISA.

### Assessment of IFN-stimulated gene expression in whole blood

Total RNA was extracted using the Paxgene blood RNA extraction (Quiagen), according to the manufacturer’s instructions. RNA concentration was assessed using a spectrophotometer (NanoDrop UV visible spectrophotometer, Thermo Fisher Scientific). Quantitative RT-PCR (qPCR) analysis was performed using the TaqMan Universal PCR Master Mix (Applied Biosystems) and complementary DNA derived from 40 ng of total RNA. Using TaqMan probes for *IFI27* (Hs01086370_m1), *IFI44L* (Hs00199115_m1), *IFIT1* (Hs00356631_g1), *ISG15* (Hs00192713_m1), *RSAD2* (Hs01057264_m1), and *SIGLEC1* (Hs00988063_m1), the relative abundance of each target transcript was normalized to the expression level of *SDHA* (Hs00188166_m1). Real-time qPCR was performed in duplicate using the LightCycler VIIA7 System (Roche). The RQ value was equal to 2^ΔΔct^ where ΔΔct is calculated by (CT target − CT SDHA) test sample − (CT target − CT SDHA) calibrator sample. ISG score was considered as the mean of the genes used to assess type I IFN.

### SARS-CoV-2 serological assay

Screening for SARS-CoV-2–specific antibodies was performed using either the SARS-CoV-2 IgG Architect (Abbott, Sligo, Ireland) following the manufacturer’s instruction or the S-Flow assay described in (*27*, *29*). Briefly, 293T-S cells stably expressing the SARS-CoV-2 Spike protein (GenBank: QHD43416.1) were generated by lentiviral transduction and selection with puromycin (1 μg/ml). Control and 293T-S cells were incubated at 4°C for 30 min with sera (1:300 dilution) in phosphate-buffered saline (PBS) containing 0.5% bovine serum albumin and 2 mM EDTA, washed with PBS, and stained using either anti-IgG Alexa Fluor 647 (dilution 1:600; Thermo Fisher Scientific), anti-IgM Alexa Fluor 488 (dilution 1:600; Thermo Fisher Scientific), or anti-IgA Alexa Fluor 647 (dilution 1:800; Jackson ImmunoResearch). Cells were washed with PBS and fixed for 10 min using 4% paraformaldehyde. Data were acquired on an Attune NxT instrument (Life Technologies) and analyzed with FlowJo 10 (BD Biosciences). Specific binding was calculated with the following formula: 100 × (% binding on 293T-Spike − % binding on control cells)/(100 − % binding on control cells).

The capacity of sera to neutralize SARS-CoV-2 was measured using lentiviral Spike pseudotypes as previously described (*27*, *29*). Briefly, 2 × 10^4^ 293T-ACE2 cells were plated in 96-well plates. Single-cycle lentiviral Spike pseudotypes encoding for a luciferase reporter gene were preincubated 30 min at RT with the serum to be tested at the indicated dilution and added to the cells. The luciferase signal was measured after 48 hours. The percentage of neutralization was calculated with the following formula, setting the “no-serum” condition at 0% and the “no-pseudotype” condition at 100%: 100 × (1 − (value with serum − value with no pseudotype)/(value with no serum − value with no pseudotype)).

### Statistical analysis

Prism (GraphPad) version 8.4.0 and R (CRAN) version 3.4.3 were used for statistical analysis. Comparisons of groups were performed using *t* tests, which are known to be robust with respect to departures from normality (*47*). Unpaired *t* test without correction was performed when no evidence for heteroscedasticity was found (*F* test of equality of variance *P* ≥ 0.2), while correction for variance inequality was performed using Welsh’s correction when *F* test *P* < 0.2. Holm-Sidak method was used to correct for multiple testing. When homoscedasticity was not clearly violated, two-way ANOVAs followed by post hoc Sidak’s procedure were also performed and yielded the same statistically significant findings. Correlations between quantitative variables were assessed using Spearman’s correlation coefficient and the associated *P* value. *P* < 0.05 was considered statistically significant.

## SUPPLEMENTARY MATERIALS

Supplementary material for this article is available at http://advances.sciencemag.org/cgi/content/full/7/34/eabg4081/DC1

## Appendix

View/request a protocol for this paper from *Bio-protocol*.

## References

[1] C. Huang, Y. Wang, X. Li, L. Ren, J. Zhao, Y. Hu, L. Zhang, G. Fan, J. Xu, X. Gu, Z. Cheng, T. Yu, J. Xia, Y. Wei, W. Wu, X. Xie, W. Yin, H. Li, M. Liu, Y. Xiao, H. Gao, L. Guo, J. Xie, G. Wang, R. Jiang, Z. Gao, Q. Jin, J. Wang, B. Cao, Clinical features of patients infected with 2019 novel coronavirus in Wuhan, China. Lancet 395, 497–506 (2020). 31986264 10.1016/S0140-6736(20)30183-5 PMC7159299

[2] H. Salje, C. T. Kiem, N. Lefrancq, N. Courtejoie, P. Bosetti, J. Paireau, A. Andronico, N. Hozé, J. Richet, C.-L. Dubost, Y. L. Strat, J. Lessler, D. Levy-Bruhl, A. Fontanet, L. Opatowski, P.-Y. Boelle, S. Cauchemez, Estimating the burden of SARS-CoV-2 in France. Science 369, 208–211 (2020). 32404476 10.1126/science.abc3517 PMC7223792

[3] M. Dai, D. Liu, M. Liu, F. Zhou, G. Li, Z. Chen, Z. Zhang, H. You, M. Wu, Q. Zheng, Y. Xiong, H. Xiong, C. Wang, C. Chen, F. Xiong, Y. Zhang, Y. Peng, S. Ge, B. Zhen, T. Yu, L. Wang, H. Wang, Y. Liu, Y. Chen, J. Mei, X. Gao, Z. Li, L. Gan, C. He, Z. Li, Y. Shi, Y. Qi, J. Yang, D. G. Tenen, L. Chai, L. A. Mucci, M. Santillana, H. Cai, Patients with cancer appear more vulnerable to SARS-CoV-2: A multicenter study during the COVID-19 outbreak. Cancer Discov. 10, 783–791 (2020). 32345594 10.1158/2159-8290.CD-20-0422 PMC7309152

[4] V. Mehta, S. Goel, R. Kabarriti, D. Cole, M. Goldfinger, A. Acuna-Villaorduna, K. Pradhan, R. Thota, S. Reissman, J. A. Sparano, B. A. Gartrell, R. V. Smith, N. Ohri, M. Garg, A. D. Racine, S. Kalnicki, R. Perez-Soler, B. Halmos, A. Verma, Case fatality rate of cancer patients with COVID-19 in a New York hospital system. Cancer Discov. 10, 935–941 (2020). 32357994 10.1158/2159-8290.CD-20-0516 PMC7334098

[5] L. Y. W. Lee, J.-B. Cazier, T. Starkey, S. E. W. Briggs, R. Arnold, V. Bisht, S. Booth, N. A. Campton, V. W. T. Cheng, G. Collins, H. M. Curley, P. Earwaker, M. W. Fittall, S. Gennatas, A. Goel, S. Hartley, D. J. Hughes, D. Kerr, A. J. X. Lee, R. J. Lee, S. M. Lee, H. Mckenzie, C. P. Middleton, N. Murugaesu, T. Newsom-Davis, A. C. Olsson-Brown, C. Palles, T. Powles, E. A. Protheroe, K. Purshouse, A. Sharma-Oates, S. Sivakumar, A. J. Smith, O. Topping, C. D. Turnbull, C. Várnai, A. D. M. Briggs, G. Middleton, R. Kerr; UK Coronavirus Cancer Monitoring Project Team, COVID-19 prevalence and mortality in patients with cancer and the effect of primary tumour subtype and patient demographics: A prospective cohort study. Lancet Oncol. 21, 1309–1316 (2020). 32853557 10.1016/S1470-2045(20)30442-3 PMC7444972

[6] L. Y. Lee, J.-B. Cazier, V. Angelis, R. Arnold, V. Bisht, N. A. Campton, J. Chackathayil, V. W. Cheng, H. M. Curley, M. W. Fittall, L. Freeman-Mills, S. Gennatas, A. Goel, S. Hartley, D. J. Hughes, D. Kerr, A. J. Lee, R. J. Lee, S. E. McGrath, C. P. Middleton, N. Murugaesu, T. Newsom-Davis, A. F. Okines, A. C. Olsson-Brown, C. Palles, Y. Pan, R. Pettengell, T. Powles, E. A. Protheroe, K. Purshouse, A. Sharma-Oates, S. Sivakumar, A. J. Smith, T. Starkey, C. D. Turnbull, C. Várnai, N. Yousaf; UK Coronavirus Monitoring Project Team, R. Kerr, G. Middleton, COVID-19 mortality in patients with cancer on chemotherapy or other anticancer treatments: A prospective cohort study. Lancet 395, 1919–1926 (2020). 32473682 10.1016/S0140-6736(20)31173-9 PMC7255715

[7] H. Miyashita, T. Mikami, N. Chopra, T. Yamada, S. Chernyavsky, D. Rizk, C. Cruz, Do patients with cancer have a poorer prognosis of COVID-19? An experience in New York City. Ann. Oncol. 31, 1088–1089 (2020). 32330541 10.1016/j.annonc.2020.04.006 PMC7172785

[8] C. Basse, S. Diakite, V. Servois, M. Frelaut, A. Noret, A. Bellesoeur, P. Moreau, M.-A. Massiani, A.-S. Bouyer, P. Vuagnat, S. Malak, F.-C. Bidard, D. Vanjak, I. Kriegel, A. Burnod, G. Bilger, T. Ramtohul, G. Dhonneur, C. Bouleuc, N. Cassoux; Institut Curie COVID Group, X. Paoletti, L. Bozec, P. Cottu, Characteristics and outcome of SARS-CoV-2 infection in cancer patients. JNCI Cancer Spectr. 5, pkaa090 (2021). 33604509 10.1093/jncics/pkaa090 PMC7665636

[9] R. S. Hotchkiss, G. Monneret, D. Payen, Immunosuppression in sepsis: A novel understanding of the disorder and a new therapeutic approach. Lancet Infect. Dis. 13, 260–268 (2013). 23427891 10.1016/S1473-3099(13)70001-X PMC3798159

[10] M. J. Delano, P. A. Ward, Sepsis-induced immune dysfunction: Can immune therapies reduce mortality? J. Clin. Invest. 126, 23–31 (2016). 26727230 10.1172/JCI82224 PMC4701539

[11] R. S. Hotchkiss, E. Colston, S. Yende, E. D. Crouser, G. S. Martin, T. Albertson, R. R. Bartz, S. C. Brakenridge, M. J. Delano, P. K. Park, M. W. Donnino, M. Tidswell, F. B. Mayr, D. C. Angus, C. M. Coopersmith, L. L. Moldawer, I. M. Catlett, I. G. Girgis, J. Ye, D. M. Grasela, Immune checkpoint inhibition in sepsis: A phase 1b randomized study to evaluate the safety, tolerability, pharmacokinetics, and pharmacodynamics of nivolumab. Intensive Care Med. 45, 1360–1371 (2019). 31576433 10.1007/s00134-019-05704-z PMC9006384

[12] H.-Y. Zheng, M. Zhang, C.-X. Yang, N. Zhang, X.-C. Wang, X.-P. Yang, X.-Q. Dong, Y.-T. Zheng, Elevated exhaustion levels and reduced functional diversity of T cells in peripheral blood may predict severe progression in COVID-19 patients. Cell. Mol. Immunol. 17, 541–543 (2020). 32203186 10.1038/s41423-020-0401-3 PMC7091621

[13] D. Weiskopf, K. S. Schmitz, M. P. Raadsen, A. Grifoni, N. M. A. Okba, H. Endeman, J. P. C. van den Akker, R. Molenkamp, M. P. G. Koopmans, E. C. M. van Gorp, B. L. Haagmans, R. L. de Swart, A. Sette, R. D. de Vries, Phenotype and kinetics of SARS-CoV-2-specific T cells in COVID-19 patients with acute respiratory distress syndrome. Sci. Immunol. 5, eabd2071 (2020). 32591408 10.1126/sciimmunol.abd2071 PMC7319493

[14] D. Mathew, J. R. Giles, A. E. Baxter, D. A. Oldridge, A. R. Greenplate, J. E. Wu, C. Alanio, L. Kuri-Cervantes, M. B. Pampena, K. D’Andrea, S. Manne, Z. Chen, Y. J. Huang, J. P. Reilly, A. R. Weisman, C. A. G. Ittner, O. Kuthuru, J. Dougherty, K. Nzingha, N. Han, J. Kim, A. Pattekar, E. C. Goodwin, E. M. Anderson, M. E. Weirick, S. Gouma, C. P. Arevalo, M. J. Bolton, F. Chen, S. F. Lacey, H. Ramage, S. Cherry, S. E. Hensley, S. A. Apostolidis, A. C. Huang, L. A. Vella; Upenn COVID Processing Unit, M. R. Betts, N. J. Meyer, E. J. Wherry, Deep immune profiling of COVID-19 patients reveals distinct immunotypes with therapeutic implications. Science 369, eabc8511 (2020). 32669297 10.1126/science.abc8511 PMC7402624

[15] A. Grifoni, D. Weiskopf, S. I. Ramirez, J. Mateus, J. M. Dan, C. R. Moderbacher, S. A. Rawlings, A. Sutherland, L. Premkumar, R. S. Jadi, D. Marrama, A. M. de Silva, A. Frazier, A. F. Carlin, J. A. Greenbaum, B. Peters, F. Krammer, D. M. Smith, S. Crotty, A. Sette, Targets of T cell responses to SARS-CoV-2 coronavirus in humans with COVID-19 disease and unexposed individuals. Cell 181, 1489–1501.e15 (2020). 32473127 10.1016/j.cell.2020.05.015 PMC7237901

[16] E. V. Robilotti, N. E. Babady, P. A. Mead, T. Rolling, R. Perez-Johnston, M. Bernardes, Y. Bogler, M. Caldararo, C. J. Figueroa, M. S. Glickman, A. Joanow, A. Kaltsas, Y. J. Lee, A. Lucca, A. Mariano, S. Morjaria, T. Nawar, G. A. Papanicolaou, J. Predmore, G. Redelman-Sidi, E. Schmidt, S. K. Seo, K. Sepkowitz, M. K. Shah, J. D. Wolchok, T. M. Hohl, Y. Taur, M. Kamboj, Determinants of COVID-19 disease severity in patients with cancer. Nat. Med. 26, 1218–1223 (2020). 32581323 10.1038/s41591-020-0979-0 PMC7785283

[17] J. Luo, H. Rizvi, J. V. Egger, I. R. Preeshagul, J. D. Wolchok, M. D. Hellmann, Impact of PD-1 blockade on severity of COVID-19 in patients with lung cancers. Cancer Discov. 10, 1121–1128 (2020). 32398243 10.1158/2159-8290.CD-20-0596 PMC7416461

[18] M. Tagliamento, F. Spagnolo, F. Poggio, D. Soldato, B. Conte, T. Ruelle, E. Barisione, A. D. Maria, L. D. Mastro, M. D. Maio, M. Lambertini, Italian survey on managing immune checkpoint inhibitors in oncology during COVID-19 outbreak. Eur. J. Clin. Invest. 50, e13315 (2020). 32535890 10.1111/eci.13315 PMC7323025

[19] O. J. Pickles, L. Y. W. Lee, T. Starkey, L. Freeman-Mills, A. Olsson-Brown, V. Cheng, D. J. Hughes, A. Lee, K. Purshouse, G. Middleton, Immune checkpoint blockade: Releasing the breaks or a protective barrier to COVID-19 severe acute respiratory syndrome? Br. J. Cancer 123, 691–693 (2020). 32546835 10.1038/s41416-020-0930-7 PMC7296191

[20] J. Hadjadj, N. Yatim, L. Barnabei, A. Corneau, J. Boussier, N. Smith, H. Péré, B. Charbit, V. Bondet, C. Chenevier-Gobeaux, P. Breillat, N. Carlier, R. Gauzit, C. Morbieu, F. Pène, N. Marin, N. Roche, T.-A. Szwebel, S. H. Merkling, J.-M. Treluyer, D. Veyer, L. Mouthon, C. Blanc, P.-L. Tharaux, F. Rozenberg, A. Fischer, D. Duffy, F. Rieux-Laucat, S. Kernéis, B. Terrier, Impaired type I interferon activity and inflammatory responses in severe COVID-19 patients. Science 369, 718–724 (2020). 32661059 10.1126/science.abc6027 PMC7402632

[21] K. Ochiai, M. Maienschein-Cline, G. Simonetti, J. Chen, R. Rosenthal, R. Brink, A. S. Chong, U. Klein, A. R. Dinner, H. Singh, R. Sciammas, Transcriptional regulation of germinal center B and plasma cell fates by dynamical control of IRF4. Immunity 38, 918–929 (2013). 23684984 10.1016/j.immuni.2013.04.009 PMC3690549

[22] D. T. Avery, S. L. Kalled, J. I. Ellyard, C. Ambrose, S. A. Bixler, M. Thien, R. Brink, F. Mackay, P. D. Hodgkin, S. G. Tangye, BAFF selectively enhances the survival of plasmablasts generated from human memory B cells. J. Clin. Invest. 112, 286–297 (2003). 12865416 10.1172/JCI18025 PMC164292

[23] P. Bastard, L. B. Rosen, Q. Zhang, E. Michailidis, H.-H. Hoffmann, Y. Zhang, K. Dorgham, Q. Philippot, J. Rosain, V. Béziat, J. Manry, E. Shaw, L. Haljasmägi, P. Peterson, L. Lorenzo, L. Bizien, S. Trouillet-Assant, K. Dobbs, A. A. de Jesus, A. Belot, A. Kallaste, E. Catherinot, Y. Tandjaoui-Lambiotte, J. L. Pen, G. Kerner, B. Bigio, Y. Seeleuthner, R. Yang, A. Bolze, A. N. Spaan, O. M. Delmonte, M. S. Abers, A. Aiuti, G. Casari, V. Lampasona, L. Piemonti, F. Ciceri, K. Bilguvar, R. P. Lifton, M. Vasse, D. M. Smadja, M. Migaud, J. Hadjadj, B. Terrier, D. Duffy, L. Quintana-Murci, D. van de Beek, L. Roussel, D. C. Vinh, S. G. Tangye, F. Haerynck, D. Dalmau, J. Martinez-Picado, P. Brodin, M. C. Nussenzweig, S. Boisson-Dupuis, C. Rodríguez-Gallego, G. Vogt, T. H. Mogensen, A. J. Oler, J. Gu, P. D. Burbelo, J. I. Cohen, A. Biondi, L. R. Bettini, M. D’Angio, P. Bonfanti, P. Rossignol, J. Mayaux, F. Rieux-Laucat, E. S. Husebye, F. Fusco, M. V. Ursini, L. Imberti, A. Sottini, S. Paghera, E. Quiros-Roldan, C. Rossi, R. Castagnoli, D. Montagna, A. Licari, G. L. Marseglia, X. Duval, J. Ghosn; HGID Lab; NIAID-USUHS Immune Response to COVID Group; COVID Clinicians; COVID-STORM Clinicians; Imagine COVID Group; French COVID Cohort Study Group; Milieu Intérieur Consortium; CoV-Contact Cohort; Amsterdam UMC Covid-19 Biobank; COVID Human Genetic Effort, J. S. Tsang, R. Goldbach-Mansky, K. Kisand, M. S. Lionakis, A. Puel, S.-Y. Zhang, S. M. Holland, G. Gorochov, E. Jouanguy, C. M. Rice, A. Cobat, L. D. Notarangelo, L. Abel, H. C. Su, J.-L. Casanova, Autoantibodies against type I IFNs in patients with life-threatening COVID-19. Science 370, eabd4585 (2020). 32972996 10.1126/science.abd4585 PMC7857397

[24] Q. Zhang, P. Bastard, Z. Liu, J. L. Pen, M. Moncada-Velez, J. Chen, M. Ogishi, I. K. D. Sabli, S. Hodeib, C. Korol, J. Rosain, K. Bilguvar, J. Ye, A. Bolze, B. Bigio, R. Yang, A. A. Arias, Q. Zhou, Y. Zhang, F. Onodi, S. Korniotis, L. Karpf, Q. Philippot, M. Chbihi, L. Bonnet-Madin, K. Dorgham, N. Smith, W. M. Schneider, B. S. Razooky, H.-H. Hoffmann, E. Michailidis, L. Moens, J. E. Han, L. Lorenzo, L. Bizien, P. Meade, A.-L. Neehus, A. C. Ugurbil, A. Corneau, G. Kerner, P. Zhang, F. Rapaport, Y. Seeleuthner, J. Manry, C. Masson, Y. Schmitt, A. Schlüter, T. L. Voyer, T. Khan, J. Li, J. Fellay, L. Roussel, M. Shahrooei, M. F. Alosaimi, D. Mansouri, H. Al-Saud, F. Al-Mulla, F. Almourfi, S. Z. Al-Muhsen, F. Alsohime, S. A. Turki, R. Hasanato, D. van de Beek, A. Biondi, L. R. Bettini, M. D’Angio, P. Bonfanti, L. Imberti, A. Sottini, S. Paghera, E. Quiros-Roldan, C. Rossi, A. J. Oler, M. F. Tompkins, C. Alba, I. Vandernoot, J.-C. Goffard, G. Smits, I. Migeotte, F. Haerynck, P. Soler-Palacin, A. Martin-Nalda, R. Colobran, P.-E. Morange, S. Keles, F. Çölkesen, T. Ozcelik, K. K. Yasar, S. Senoglu, Ş. N. Karabela, C. Rodríguez-Gallego, G. Novelli, S. Hraiech, Y. Tandjaoui-Lambiotte, X. Duval, C. Laouénan; COVID-STORM Clinicians; COVID Clinicians; Imagine COVID Group; French COVID Cohort Study Group; CoV-Contact Cohort; Amsterdam UMC Covid-19 Biobank; COVID Human Genetic Effort; NIAID-USUHS/TAGC COVID Immunity Group, A. L. Snow, C. L. Dalgard, J. D. Milner, D. C. Vinh, T. H. Mogensen, N. Marr, A. N. Spaan, B. Boisson, S. Boisson-Dupuis, J. Bustamante, A. Puel, M. J. Ciancanelli, I. Meyts, T. Maniatis, V. Soumelis, A. Amara, M. Nussenzweig, A. García-Sastre, F. Krammer, A. Pujol, D. Duffy, R. P. Lifton, S.-Y. Zhang, G. Gorochov, V. Béziat, E. Jouanguy, V. Sancho-Shimizu, C. M. Rice, L. Abel, L. D. Notarangelo, A. Cobat, H. C. Su, J.-L. Casanova, Inborn errors of type I IFN immunity in patients with life-threatening COVID-19. Science 370, eabd4570 (2020). 32972995 10.1126/science.abd4570 PMC7857407

[25] D. S. Stephens, M. J. McElrath, COVID-19 and the path to immunity. JAMA 324, 1279–1281 (2020). 32915201 10.1001/jama.2020.16656 PMC12177933

[26] Y. Peng, A. J. Mentzer, G. Liu, X. Yao, Z. Yin, D. Dong, W. Dejnirattisai, T. Rostron, P. Supasa, C. Liu, C. López-Camacho, J. Slon-Campos, Y. Zhao, D. I. Stuart, G. C. Paesen, J. M. Grimes, A. A. Antson, O. W. Bayfield, D. E. D. P. Hawkins, D.-S. Ker, B. Wang, L. Turtle, K. Subramaniam, P. Thomson, P. Zhang, C. Dold, J. Ratcliff, P. Simmonds, T. de Silva, P. Sopp, D. Wellington, U. Rajapaksa, Y.-L. Chen, M. Salio, G. Napolitani, W. Paes, P. Borrow, B. M. Kessler, J. W. Fry, N. F. Schwabe, M. G. Semple, J. K. Baillie, S. C. Moore, P. J. M. Openshaw, M. A. Ansari, S. Dunachie, E. Barnes, J. Frater, G. Kerr, P. Goulder, T. Lockett, R. Levin, Y. Zhang, R. Jing, L.-P. Ho; Oxford Immunology Network Covid-19 Response T cell Consortium; ISARIC4C Investigators, R. J. Cornall, C. P. Conlon, P. Klenerman, G. R. Screaton, J. Mongkolsapaya, A. McMichael, J. C. Knight, G. Ogg, T. Dong, Broad and strong memory CD4^+^ and CD8^+^ T cells induced by SARS-CoV-2 in UK convalescent individuals following COVID-19. Nat. Immunol. 21, 1336–1345 (2020). 32887977 10.1038/s41590-020-0782-6 PMC7611020

[27] L. Grzelak, S. Temmam, C. Planchais, C. Demeret, L. Tondeur, C. Huon, F. Guivel-Benhassine, I. Staropoli, M. Chazal, J. Dufloo, D. Planas, J. Buchrieser, M. M. Rajah, R. Robinot, F. Porrot, M. Albert, K.-Y. Chen, B. Crescenzo-Chaigne, F. Donati, F. Anna, P. Souque, M. Gransagne, J. Bellalou, M. Nowakowski, M. Backovic, L. Bouadma, L. L. Fevre, Q. L. Hingrat, D. Descamps, A. Pourbaix, C. Laouénan, J. Ghosn, Y. Yazdanpanah, C. Besombes, N. Jolly, S. Pellerin-Fernandes, O. Cheny, M.-N. Ungeheuer, G. Mellon, P. Morel, S. Rolland, F. A. Rey, S. Behillil, V. Enouf, A. Lemaitre, M.-A. Créach, S. Petres, N. Escriou, P. Charneau, A. Fontanet, B. Hoen, T. Bruel, M. Eloit, H. Mouquet, O. Schwartz, S. van der Werf, A comparison of four serological assays for detecting anti–SARS-CoV-2 antibodies in human serum samples from different populations. Sci. Transl. Med. 12, eabc3103 (2020). 32817357 10.1126/scitranslmed.abc3103 PMC7665313

[28] L. Grzelak, A. Velay, Y. Madec, F. Gallais, I. Staropoli, C. Schmidt-Mutter, M.-J. Wendling, N. Meyer, C. Planchais, D. Rey, H. Mouquet, N. Reix, L. Glady, Y. Hansmann, T. Bruel, J. De Séze, A. Fontanet, M. Gonzalez, O. Schwartz, S. Fafi-Kremer, Sex differences in the evolution of neutralizing antibodies to SARS-CoV-2. J. Infect. Dis. 2021, jiab127 (2021). 10.1093/infdis/jiab127 PMC7989436 33693749

[29] J. Dufloo, L. Grzelak, I. Staropoli, Y. Madec, L. Tondeur, F. Anna, S. Pelleau, A. Wiedemann, C. Planchais, J. Buchrieser, R. Robinot, M.-N. Ungeheuer, H. Mouquet, P. Charneau, M. White, Y. Lévy, B. Hoen, A. Fontanet, O. Schwartz, T. Bruel, Asymptomatic and symptomatic SARS-CoV-2 infections elicit polyfunctional antibodies. Cell. Rep. Med. 2, 100275 (2021). 33899033 10.1016/j.xcrm.2021.100275 PMC8057765

[30] C. Trojaniello, M. G. Vitale, P. A. Ascierto, Checkpoint inhibitor therapy for skin cancer may be safe in patients with asymptomatic COVID-19. Ann. Oncol. 32, 674–676 (2021). 33600920 10.1016/j.annonc.2021.02.008 PMC7884251

[31] J. Attanasio, E. J. Wherry, Costimulatory and coinhibitory receptor pathways in Infectious Disease. Immunity 44, 1052–1068 (2016). 27192569 10.1016/j.immuni.2016.04.022 PMC4873956

[32] D. L. Barber, E. J. Wherry, D. Masopust, B. Zhu, J. P. Allison, A. H. Sharpe, G. J. Freeman, R. Ahmed, Restoring function in exhausted CD8 T cells during chronic viral infection. Nature 439, 682–687 (2006). 16382236 10.1038/nature04444

[33] P. M. Odorizzi, K. E. Pauken, M. A. Paley, A. Sharpe, E. J. Wherry, Genetic absence of PD-1 promotes accumulation of terminally differentiated exhausted CD8^+^ T cells. J. Exp. Med. 212, 1125–1137 (2015). 26034050 10.1084/jem.20142237 PMC4493417

[34] L. Trautmann, L. Janbazian, N. Chomont, E. A. Said, S. Gimmig, B. Bessette, M.-R. Boulassel, E. Delwart, H. Sepulveda, R. S. Balderas, J.-P. Routy, E. K. Haddad, R.-P. Sekaly, Upregulation of PD-1 expression on HIV-specific CD8^+^ T cells leads to reversible immune dysfunction. Nat. Med. 12, 1198–1202 (2006). 16917489 10.1038/nm1482

[35] J. J. Erickson, P. Gilchuk, A. K. Hastings, S. J. Tollefson, M. Johnson, M. B. Downing, K. L. Boyd, J. E. Johnson, A. S. Kim, S. Joyce, J. V. Williams, Viral acute lower respiratory infections impair CD8^+^ T cells through PD-1. J. Clin. Invest. 122, 2967–2982 (2012). 22797302 10.1172/JCI62860 PMC3408742

[36] K. E. Remy, M. Mazer, D. A. Striker, A. H. Ellebedy, A. H. Walton, J. Unsinger, T. M. Blood, P. A. Mudd, D. J. Yi, D. A. Mannion, D. F. Osborne, R. S. Martin, N. J. Anand, J. P. Bosanquet, J. Blood, A. M. Drewry, C. C. Caldwell, I. R. Turnbull, S. C. Brakenridge, L. L. Moldwawer, R. S. Hotchkiss, Severe immunosuppression and not a cytokine storm characterizes COVID-19 infections. JCI Insight 5, e140329 (2020). 10.1172/jci.insight.140329 PMC7526441 32687484

[37] M. Kox, N. J. B. Waalders, E. J. Kooistra, J. Gerretsen, P. Pickkers, Cytokine levels in critically ill patients with COVID-19 and other conditions. JAMA 324, 1565 (2020). 10.1001/jama.2020.17052 PMC7489366 32880615

[38] D. E. Leisman, L. Ronner, R. Pinotti, M. D. Taylor, P. Sinha, C. S. Calfee, A. V. Hirayama, F. Mastroiani, C. J. Turtle, M. O. Harhay, M. Legrand, C. S. Deutschman, Cytokine elevation in severe and critical COVID-19: A rapid systematic review, meta-analysis, and comparison with other inflammatory syndromes. Lancet Respir. Med. 8, 1233–1244 (2020). 33075298 10.1016/S2213-2600(20)30404-5 PMC7567529

[39] R. Zhou, K. K.-W. To, Y.-C. Wong, L. Liu, B. Zhou, X. Li, H. Huang, Y. Mo, T.-Y. Luk, T. T.-K. Lau, P. Yeung, W.-M. Chan, A. K.-L. Wu, K.-C. Lung, O. T.-Y. Tsang, W.-S. Leung, I. F.-N. Hung, K.-Y. Yuen, Z. Chen, Acute SARS-CoV-2 infection impairs dendritic cell and T cell responses. Immunity 53, 864–877.e5 (2020). 32791036 10.1016/j.immuni.2020.07.026 PMC7402670

[40] J. R. Habel, T. H. O. Nguyen, C. E. van de Sandt, J. A. Juno, P. Chaurasia, K. Wragg, M. Koutsakos, L. Hensen, X. Jia, B. Chua, W. Zhang, H.-X. Tan, K. L. Flanagan, D. L. Doolan, J. Torresi, W. Chen, L. M. Wakim, A. C. Cheng, P. C. Doherty, J. Petersen, J. Rossjohn, A. K. Wheatley, S. J. Kent, L. C. Rowntree, K. Kedzierska, Suboptimal SARS-CoV-2−specific CD8^+^ T cell response associated with the prominent HLA-A*02:01 phenotype. Proc. Natl. Acad. Sci. U.S.A 117, 24384–24391 (2020). 32913053 10.1073/pnas.2015486117 PMC7533701

[41] J. Shi, S. Hou, Q. Fang, X. Liu, X. Liu, H. Qi, PD-1 controls follicular T helper cell positioning and function. Immunity 49, 264–274.e4 (2018). 30076099 10.1016/j.immuni.2018.06.012 PMC6104813

[42] M. C. Woodruff, R. P. Ramonell, D. C. Nguyen, K. S. Cashman, A. S. Saini, N. S. Haddad, A. M. Ley, S. Kyu, J. C. Howell, T. Ozturk, S. Lee, N. Suryadevara, J. B. Case, R. Bugrovsky, W. Chen, J. Estrada, A. Morrison-Porter, A. Derrico, F. A. Anam, M. Sharma, H. M. Wu, S. N. Le, S. A. Jenks, C. M. Tipton, B. Staitieh, J. L. Daiss, E. Ghosn, M. S. Diamond, R. H. Carnahan, J. E. Crowe, W. T. Hu, F. E.-H. Lee, I. Sanz, Extrafollicular B cell responses correlate with neutralizing antibodies and morbidity in COVID-19. Nat. Immunol. 21, 1506–1516 (2020). 33028979 10.1038/s41590-020-00814-z PMC7739702

[43] H. S.-C. Wong, C.-L. Guo, G.-H. Lin, K.-Y. Lee, Y. Okada, W.-C. Chang, Transcriptome network analyses in human coronavirus infections suggest a rational use of immunomodulatory drugs for COVID-19 therapy. Genomics 113, 564–575 (2021). 33482326 10.1016/j.ygeno.2020.12.041 PMC7817445

[44] B. Waissengrin, A. Agbarya, E. Safadi, H. Padova, I. Wolf, Short-term safety of the BNT162b2 mRNA COVID-19 vaccine in patients with cancer treated with immune checkpoint inhibitors. Lancet Oncol. 22, 581–583 (2021). 33812495 10.1016/S1470-2045(21)00155-8 PMC8016402

[45] D. M. Smadja, C. L. Guerin, R. Chocron, N. Yatim, J. Boussier, N. Gendron, L. Khider, J. Hadjadj, G. Goudot, B. Debuc, P. Juvin, C. Hauw-Berlemont, J.-L. Augy, N. Peron, E. Messas, B. Planquette, O. Sanchez, B. Charbit, P. Gaussem, D. Duffy, B. Terrier, T. Mirault, J.-L. Diehl, Angiopoietin-2 as a marker of endothelial activation is a good predictor factor for intensive care unit admission of COVID-19 patients. Angiogenesis 23, 611–620 (2020). 32458111 10.1007/s10456-020-09730-0 PMC7250589

[46] J. Vandesompele, K. De Preter, F. Pattyn, B. Poppe, N. Van Roy, A. De Paepe, F. Speleman, Accurate normalization of real-time quantitative RT-PCR data by geometric averaging of multiple internal control genes. Genome Biol. 3, RESEARCH0034 (2002). 12184808 10.1186/gb-2002-3-7-research0034 PMC126239

[47] H. O. Posten, Robustness of the Two-Sample T-Test, in *Robustness of Statistical Methods and Nonparametric Statistics*, D. Rasch, M. L. Tiku, Eds. (Theory and Decision Library, Springer, 1984), pp. 92–99.

